# Outcomes of active cervical therapeutic exercise on dynamic intervertebral foramen changes in neck pain patients with disc herniation

**DOI:** 10.1186/s12891-022-05670-6

**Published:** 2022-07-30

**Authors:** Shyi-Kuen Wu, Han-Yu Chen, Jia-Yuan You, Jian-Guo Bau, Yu-Chen Lin, Li-Chieh Kuo

**Affiliations:** 1 Department of Physical Therapy, HungKuang University, Taichung, Taiwan; 2 Department of Physical Therapy, I-Shou University, Kaohsiung, Taiwan; 3 Department of Biomedical Engineering, HungKuang University, Taichung, Taiwan; 4 Department of Occupational Therapy, College of Medicine, National Cheng Kung University, Tainan, Taiwan; 5 Medical Device Innovation Center, National Cheng Kung University, Tainan, Taiwan

**Keywords:** Disc herniation, Active therapeutic exercise, Intervertebral foramen, Videofluoroscopy

## Abstract

**Background:**

To better understand biomechanical factors that affect intervertebral alignment throughout active therapeutic exercise, it is necessary to determine spinal kinematics when subjects perform spinal exercises. This study aims to investigate the outcomes of active cervical therapeutic exercise on intervertebral foramen changes in neck pain patients with disc herniation.

**Methods:**

Thirty diagnosed C4/5 and/or C5/6 disc-herniated patients receiving an 8-week cervical therapeutic exercise program were followed up with videofluoroscopic images. The dynamic changes in the foramen were computed at different timepoints, including the neutral position, end-range positions in cervical flexion-extension, protrusion-retraction, and lateral flexion movements.

**Results:**

The results showed that the active cervical flexion, retraction, and lateral flexion away from the affected side movements increased the area of the patients’ intervertebral foramen; while the active extension, protrusion, and lateral flexion toward the affected side reduced the areas of intervertebral foramen before treatment. After the treatment, the active cervical flexion significantly increased the C2/3, C3/4, and C6/7 foramen area by 5.02–8.67% (*p* = 0.001 ~ 0.029), and the extension exercise significantly reduced the C2/3 and C4/5 area by 5.12–9.18% (*p* = 0.001 ~ 0.006) compared to the baseline. Active retraction movement significantly increased the foramen area from C2/3 to C6/7 by 3.82–8.66% (*p* = 0.002 ~ 0.036 with exception of C5/6). Active lateral flexion away from the affected side significantly increased the foramen by 3.71–6.78% (*p* = 0.007 ~ 0.046 with exception of C6/7).

**Conclusions:**

The 8-week therapeutic exercises including repeated cervical retraction, extension, and lateral flexion movements to the lesion led to significant changes and improvements in intervertebral foramen areas of the patients with disc herniation.

**Trial registration:**

ISRCTN61539024

## Background

Deviations from neutral alignment of human body segments such as the head, neck, and trunk are commonly identified as excessive curvature or reductions in curvature [1]. The cervical spine supports the skull and allows for a wide range of physiological neck motion to interact with various daily or occupational performances. Work-related neck and upper limb disorders have been reported to be associated with long hours of computer work and prolonged periods of holding a static posture that might easily lead to deviation in body alignment [2, 3]. Long-term stature-like posture is most pronounced in the neck and shoulder region, resulting in increased forward neck flexion posture and elevated static muscle tension in the region. The consequences of increased forward neck flexion, including increased tension in the postural stabilizing muscles as well as increased compressive forces in the articulations of the cervical spine, further cause higher risk of neck and upper back pain [3]. Although all muscles contribute to the support of the spine in a complex way, previous research indicated that patients with neck and back pain disorders alter the muscular morphology and motor control of these muscles [3]. The static neck and shoulder posture has been frequently assumed as a possible risk factor in work-related neck and upper limb disorders among office workers. An association linking prolonged static posture with increased muscle loading and a subsequent increased risk for developing symptoms in the neck and upper back has been reported [1, 4]. The cumulative effects of these working conditions demanding static and deviated posture may greatly contribute to the development of musculoskeletal discomfort, such as neck pain.

Neck pain affects around half of general population at some time during their lifespan and results in mild to severe disability [2, 3]. The majority of acute episodes of spinal pain are mechanical, postulated to arise from an injury to or derangement of some structure within the spine. These patients typically suffer in the neck, shoulder blades or from radiating arm symptoms such as pain, sensory disorders, reflex abnormalities, and motor weakness [1, 3, 4]. However, the pathogenesis of neck pain related to alterations in cervical biomechanics or changes in morphology have not been completely explored. The intervertebral disc plays a role in dampening of the compressive loads experienced when engaging in various daily and occupational activities [5, 6]. When the disc is herniated and degenerated, this can lead to mechanical compression or chemical irritation of the nerve root, which causes neurological deficits and corresponding symptoms [7, 8]. A herniated intervertebral disc is usually believed to cause compression of a spinal nerve within an intervertebral foramen, commonly clinically known as foraminal stenosis of the spine [8–10]. A herniated disc with irritation of spinal nerves is a common neck or back condition that cause neck or back and limb pain. Several reports have indicated that highly repetitive flexion motions result in intervertebral disc herniation [11, 12].

Although a radiographic evaluation on the sagittal plane is feasible for the assessment of disc degeneration, the motion profiles of a spine with a herniated disc are not easily addressed in routine examinations. Cervical disc herniation or other space-occupying lesions commonly result in nerve root inflammation, impingement, or both [10]. Although disc herniation may be attributed to biomechanical changes in segmental movement, disc mechanism, and foramen morphology, lacking in dynamic investigations regarding the movement or anatomic features of the cervical spine obscure further understanding of cervical biomechanics in disc-herniated patients. The size and shape of the intervertebral foramen and their spatial relationship with the nerve root is important. Variations in the size and shape of the foramen are often associated with symptoms of nerve root compression in the spinal region. These changes in foraminal dimensions have been commonly used to understand patho-anatomy with respect to corresponding changes in symptoms, clinical signs, or the pathophysiology of some spinal disorders [13, 14].

Exercise is recommended in patients with radicular pain due to disc herniation. Clinical efficacies including pain reduction and functional improvements have been demonstrated [15]. Active spinal exercises are one type of nonsurgical interventional procedure commonly used for treatment of chronic neck disorders [16, 17]. Physical therapists frequently instruct patients with active therapeutic exercises, such as cervical retraction, extension, and lateral flexion, to treat spinal dysfunctions [15–17]. However, the influences of positional changes in the cervical spine on the dimensions of the intervertebral foramen are not well documented in the literature.

Long and Donelson [18] conducted a randomized control trial consisting of therapeutic exercise for patients with spinal pain. Their findings indicated the exercises matching the subjects’ directional preference significantly and rapidly decreased pain and medication use, and led to improvements in the functional outcomes. Specific spinal exercises have been recommended, but their effects on and relationship to intervertebral posture remain unclear. In order to better understand the biomechanical factors that affect spinal alignment throughout active therapeutic exercise, it is necessary to determine the spinal kinematics when subjects perform spinal exercises. Therefore, the purpose of this study is to analyze the outcomes of active cervical therapeutic exercises on changes in the intervertebral foramen after therapeutic exercise treatment sessions in patients with disc herniation. It was hypothesized that 1) the areas of the intervertebral foramen may be changed based on different cervical spinal postures and movements, and 2) an 8-week intervention consisting of active spinal exercises utilizing different movement patterns may result in significant changes in the intervertebral foramen.

## Methods

### Study design

This was a one-group pretest-posttest case series study design with pre-treatment and 8-week follow-up assessments. This clinical trial has been registered under the ISRCTN registry with the registration number: ISRCTN61539024 and the date of first registration on 02/08/2016 (retrospectively registered; 10.1186/ISRCTN61539024).

### Participants

Thirty diagnosed C4/5 and/or C5/6 disc-herniated patients with neck, shoulder blade, or radiating arm symptoms referred from the Department of Physical Medicine and Rehabilitation of a medical center in middle Taiwan were recruited to participate in this study. Their symptom duration was 3.4 ± 2.1 months and pain or numbness locations were neck (83%), shoulder girdle (67%), interscapular (60%) and forearm (33%) areas. The disc herniation pathology was diagnosed by the clinical presentations in symptoms and signs or medical images of these patients. The patients with disc herniation have intermittent pain or numbness over the neck and upper extremities and they demonstrated a limited range of neck motion. The exclusion criteria for the participants were as follows: (1) past history of cervical surgery, such as disc replacement, bone fusion, discectomy, etc., (2) significant potential for spinal cord injury, such as cord impingement from a large disk herniation, (3) advanced cervical spondylosis, (4) severe spinal stenosis, (5) inflammatory arthritic disorders (ankylosing spondylitis or rheumatoid arthritis), (6) severe spinal instability, and (7) pregnancy [19–22]. The Visual Analogue Scale (VAS) and Neck Disability Index (NDI) were applied to evaluate the patient’s status presence and to assess the evolution across the intervention in this study.

### Ethics statement

The study was reviewed and approved by the Research Ethical Committee of the China Medical University & Hospital, Taichung, Taiwan. (reference #: CRREC-105-011). Prior to participation, each participant was informed about the study aims and experimental procedures and then was asked to sign a written consent form.

### Interventions

**Fig. 1:**
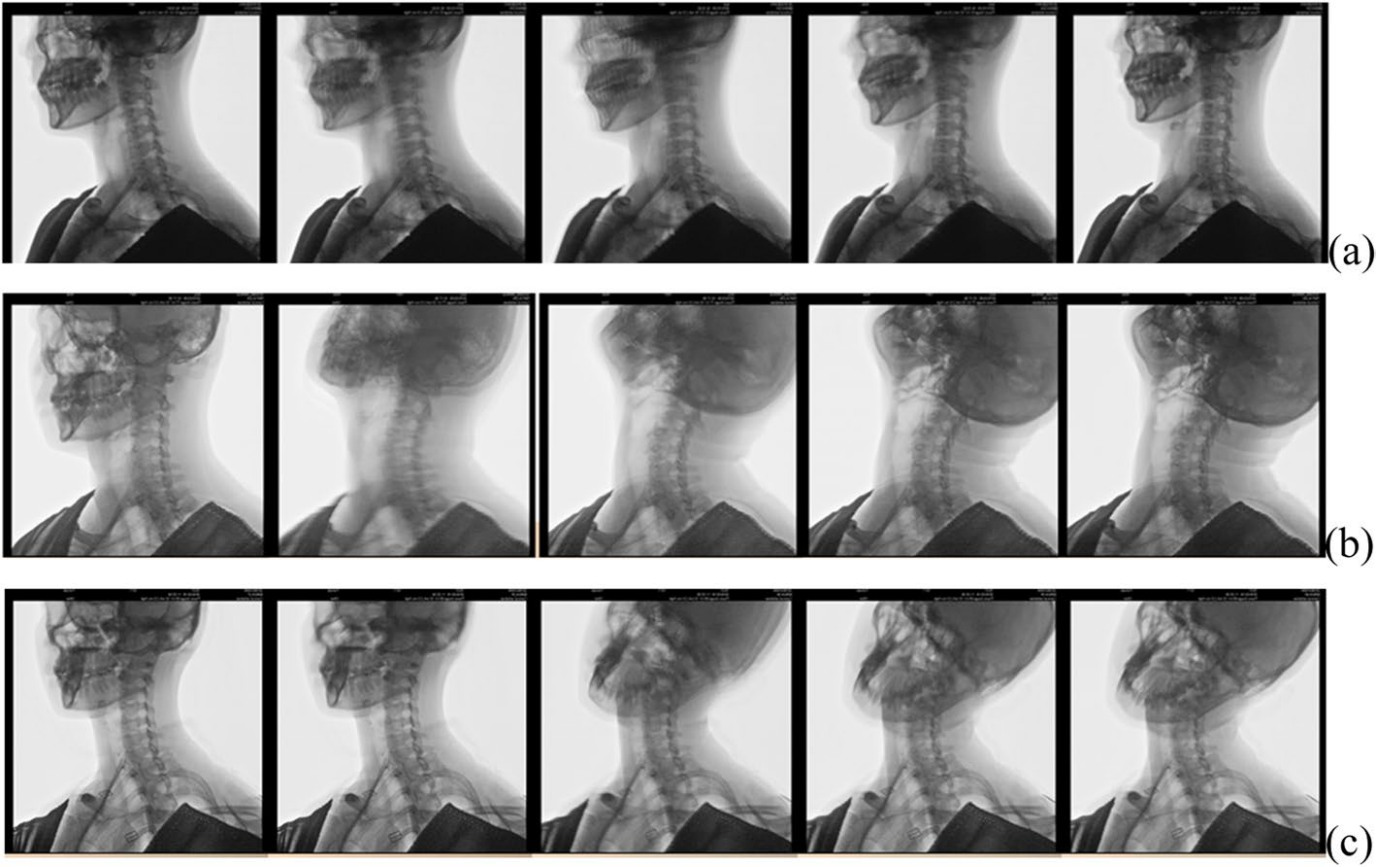
The spinal therapeutic exercises mainly included end-range repeated cervical retraction (**a**), extension (**b**), and lateral flexion (**c**) movements toward the lesion in order to reduce the nucleus displacement and peripherization inside the disc

Active spinal exercise is a nonsurgical interventional procedure used for treatment of chronic neck and back disorders. The spinal approach postulated by McKenzie indicated that compensatory pressure on the disc in the direction of the lesion can reduce the displacement of the nucleus in the disc [16, 18, 19]. The 8-week spinal therapeutic exercises in the present study mainly included end-range repeated cervical retraction, extension, and lateral flexion movements to the lesion in order to reduce the nucleus displacement and peripheralization inside the disc (Fig. 1).

The active exercise was suggested to be performed 10–15 times each session, 6–10 sessions per day [18, 22–24]. The programs were administrated by an experienced orthopedic physical therapist in the Department of Physical Medicine and Rehabilitation to match the patients’ directional preferences, where significantly and rapidly decreased pain and improved functional outcomes were reported. The neck movement was performed to the end range with less than 6/10 in visual analogue scale and without symptom peripheralization.

### Experimental setup and procedures

The videofluoroscopic system (Toshiba, Tokyo, Japan) in the Department of Radiology of this medical center was used to record continuous segmental movement of the cervical spine at a rate of 30 frames per second. During preparation and screening, the images were displayed on a high-resolution monitor and recorded with a digital camcorder. The radiographic beam field of the videofluoroscopy unit was collimated to obtain optimal image sharpness. The size of the imaging field was also adjusted to view the entire alignment of the cervical spine posture through the range of movement.

The participants were requested to wear X-ray protective clothing and to stand between the image intensifier and the examination table. They were instructed on how to perform the therapeutic exercises for the cervical spine. The measurement of intervertebral posture and positional biomechanics in this study involved two assumptions: First, active movement has been reported to be accompanied by a limited range of motion in different motion planes [24]. Second, the vertebral body was assumed to be a rigid body for the purposes of the kinematic analysis. Before actual screening, the participants were allowed to practice the active cervical therapeutic exercises a few times with correction to reduce trunk and out-of-plane motions. The subjects could visualize their neck motions on the display of a widescreen liquid crystal display throughout the movements.

The recorded video images of the spinal segment motion were captured at 30 frames/ second using the input/output and monitoring solution, Avid Mojo (Avid Inc., USA). The digital images were then transformed into sequences of bitmap pictures with the aid of Canopus Edius computer software (Canopus Co., Ltd., USA). Ten pictures in evenly divided intervals of each cervical therapeutic exercise were selected for digitizing, respectively.

**Fig. 2:**
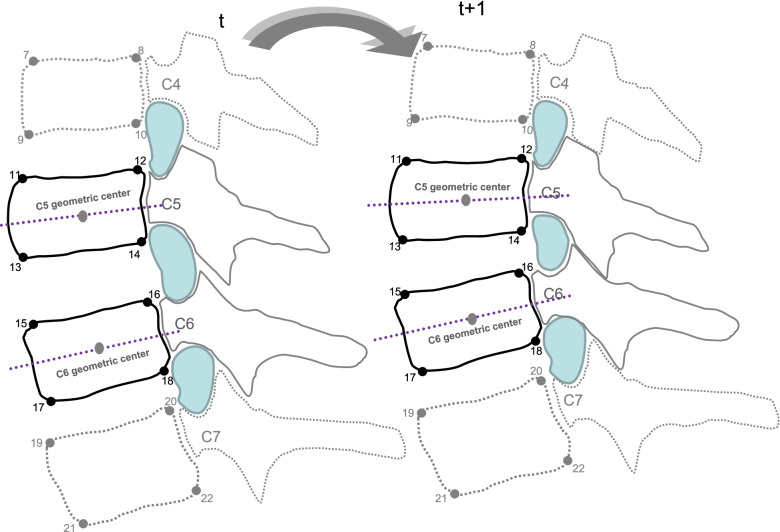
Identification of bony landmarks and segmental neural foramen during therapeutic movement (cervical extension)

During the image processing, cross-sectional areas of the foramen were measured using SigmaScan 5.0 (SPSS Inc., Chicago, IL, USA) on a high-resolution monitor [25, 26] (Fig. 2). The methods for identifying the vertebral landmarks and foraminal dimensions were blinded between examiners, and a total of two sets of 1080 image sequences (30 subjects × 6 movements × 6 intervals) were digitized by two trained laboratory technicians. The cross-sectional areas of the C2/3 to C6/7 foramen were analyzed in different positions, including the neutral position, end-range positions in cervical flexion, extension, protrusion, retraction, and bilateral lateral flexion movements. The area change of intervertebral foramen in flexion was defined as the foramen difference between the neutral position and end-range position in cervical flexion. The comparison before and after the 8-week exercise interventions, the dynamic area change of intervertebral foramen in flexion was defined as the foramen differences between the neutral position and end-range position in cervical flexion before and after treatment.

### Adverse effects study report protocol

In this study, an adverse event was defined as an injury to the neck or upper limb attributable to the intervention requiring a visit to a hospital. The small degree of increased risk was stated in the informed consent sheet.

### Statistical analyses

Analyses were performed using the Scientific Package for Social Sciences (version 19; SPSS, Chicago, IL, USA). The reliability of the digitizing image procedures at 2-weeks intervals and between raters were assessed using the intraclass correlation coefficient (ICC 2,1) and standard error of measurement (SEM) methods on ten different randomly-selected subjects [27, 28]. The minimal detectable change (MDC) for the measurement error was further calculated by 1.96 × √2 × SEM [27, 28]. The comparisons among changes in the intervertebral foramens throughout the ranges of the therapeutic exercises were performed with a paired t-test with a probability level of *p* < 0.05 was selected as the criterion for significant differences across the treatment sessions.

## Results

The age of the 30 patients (14 females and 16 males) with disc herniation ranged from 20 to 50 years with a mean age of 30.5 ± 5.7 years. Their body heights and weights were 168.5 ± 6.2 cm and 66.2 ± 6.8 kgw. The ICCs for the calculated foramen area within raters and between raters during the exercises averaged 0.951 and 0.842, respectively. The SEM values of calculated foramen area ranged from 0.156 to 0.409 mm^2^ with an average of 0.276 mm^2^ (0.604%). The minimal detectable change (MDC) values of calculated foramen area ranged from 0.378 mm^2^ (0.828%) to 0.994 mm^2^ (2.174%) with an average of 0.797 mm^2^ (1.745%) (Table 1). The mean Visual Analogue Scale (VAS) was 4.7 ± 2.4 at baseline and significantly reduced to 1.8 ± 1.2 after the intervention. The mean Neck Disability Index (NDI) was 19.2 ± 4.9 at baseline and also significantly decreased to 5.9 ± 3.4 after the intervention (both *p* < 0.005). All the thirty patients with disc herniation completed the intervention program and no negative outcomes associated with attempted movement interventions found during the period of participation.

**Table 1:** The standard error of measurement (SEM) and minimal detectable change (MDC) were presented by the mean (mm^2^) and percentage (%) of the intervertebral foramen area before and after spinal therapeutic exercise movements, including cervical flexion-extension, protrusion-retraction, and lateral flexion toward or away from the affected side

**SEM**	**Flexion before treatment**	**Extension before treatment**	**Flexion after treatment**	**Extension after treatment**
Mean	0.197	0.289	0.340	0.409
%	0.431%	0.633%	0.744%	0.897%
	**Protrusion before treatment**	**Retraction before treatment**	**Protrusion after treatment**	**Retraction after treatment**
Mean	0.267	0.156	0.392	0.268
%	0.584%	0.342%	0.859%	0.587%
	**Lateral flexion toward before treatment**	**Lateral flexion away before treatment**	**Lateral flexion toward after treatment**	**Lateral flexion away after treatment**
Mean	0.203	0.208	0.272	0.309
%	0.443%	0.454%	0.595%	0.675%
Total				0.276
				0.604%
**MDC**	**Flexion before treatment**	**Extension before treatment**	**Flexion after treatment**	**Extension after treatment**
Mean	0.478	0.702	0.825	0.994
%	1.046%	1.536%	1.805%	2.174%
	**Protrusion before treatment**	**Retraction before treatment**	**Protrusion after treatment**	**Retraction after treatment**
Mean	0.648	0.378	0.952	0.650
%	1.417%	0.828%	2.083%	1.423%
	**Lateral flexion toward before treatment**	**Lateral flexion away before treatment**	**Lateral flexion toward after treatment**	**Lateral flexion away after treatment**
Mean	0.491	0.503	0.660	0.748
%	1.074%	1.101%	1.444%	1.637%
Total				0.797
				1.745%

### Cervical flexion

**Fig. 3:**
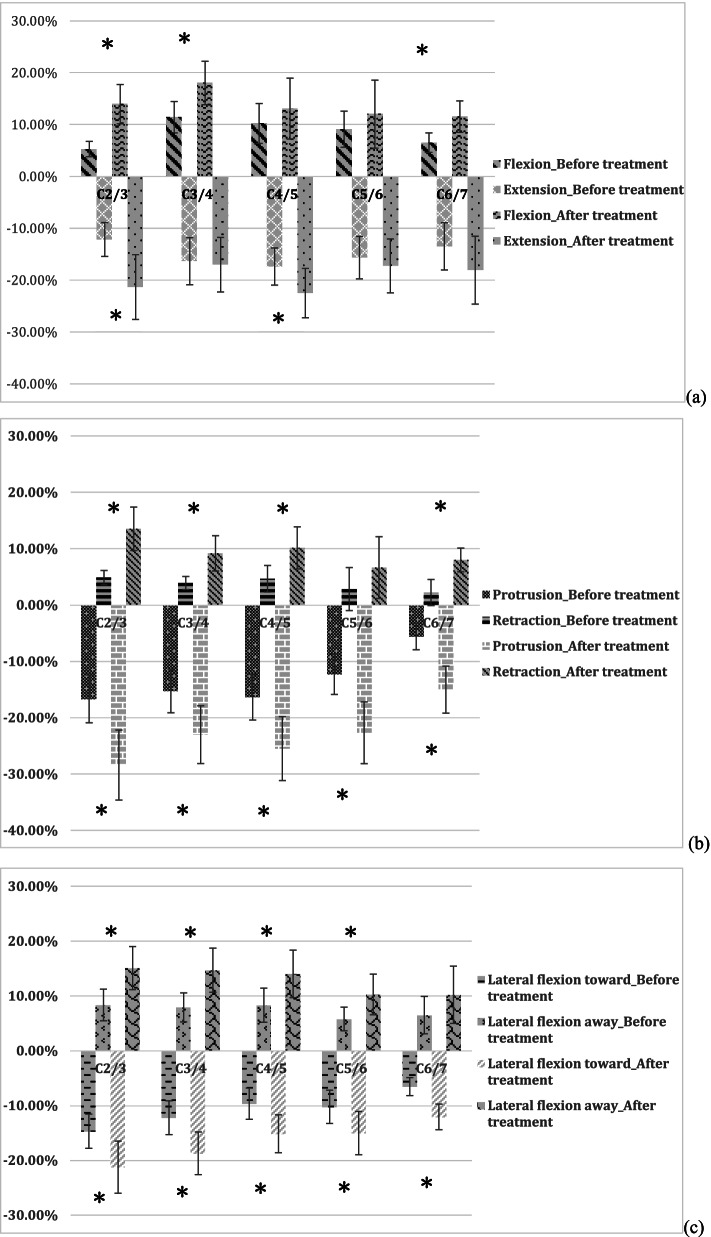
The dimensions of the intervertebral foramen before and after various spinal therapeutic exercise movements, including cervical flexion-extension (**a**), protrusion-retraction (**b**), and lateral flexion toward or away from the affected side (**c**)

The mean range of the dimensions of the intervertebral foramen during active flexion and extension are presented in Fig. 3(a). The active cervical flexion movement increased the area of intervertebral foramen from C2/3 to C6/7 by 5.29 ± 1.47%, 11.43 ± 3.02%, 10.22 ± 3.85%, 9.12 ± 3.49%, and 6.52 ± 1.88%, respectively, as compared to the neutral position in the disc-herniated patients before treatment (*p* = 0.001 ~ 0.033). After the treatment, the active cervical flexion movement increased the area of the intervertebral foramen from C2/3 to C6/7 by 13.96 ± 3.78%, 18.03 ± 4.18%, 13.09 ± 5.86%, 12.03 ± 6.56%, and 11.55 ± 3.04%, respectively, as compared to the neutral position (all *p* < 0.001). The area of the intervertebral foramen in the cervical flexion movement significantly increased after treatment, especially in C2/3 (*p* = 0.002), C3/4 (*p* = 0.001), and C6/7 (*p* = 0.029).

### Cervical extension

The extension exercise reduced the foramen area from C2/3 to C6/7 by 12.15 ± 3.26%, 16.33 ± 4.55%, 17.36 ± 3.58%, 15.66 ± 4.08%, and 13.48 ± 4.56%, respectively, as compared to the neutral position before treatment (all *p* < 0.001) (Fig. 3a). After the treatment, the extension exercise reduced the foramen area from C2/3 to C6/7 by 21.33 ± 6.25%, 17.02 ± 5.26%, 22.48 ± 4.76%, 17.24 ± 5.20%, and 18.07 ± 6.54%, respectively, as compared to the neutral position (*p* = 0.001 ~ 0.015). The area of the intervertebral foramen in terms of the cervical extension movement increased significantly after treatment in C2/3 (*p* = 0.001) and C4/5 (*p* = 0.006).

### Cervical protrusion

The mean range of the dimensions of the intervertebral foramen during active protrusion and retraction are presented in Fig. 3b. The active cervical protrusion movement decreased the area of the intervertebral foramen from C2/3 to C6/7 by 16.68 ± 4.23%, 15.26 ± 3.88%, 16.37 ± 4.06%, 12.30 ± 3.59%, and 5.62 ± 2.33%, respectively, as compared to the neutral position in the disc-herniated patients before treatment (all *p* < 0.001, except for C6/7 (*p* = 0.069)). After the treatment, the active cervical protrusion movement significantly decreased the area of the intervertebral foramen from C2/3 to C6/7 by 28.43 ± 6.22%, 23.03 ± 5.13%, 25.50 ± 5.66%, 22.69 ± 5.48%, and 15.03 ± 4.17%, respectively, as compared to the neutral position (all *p* < 0.001). For the comparison before and after therapeutic exercise sessions, the area of the intervertebral foramen in the cervical protrusion movement significantly decreased to a greater extent after treatment (*p* = 0.001 ~ 0.004).

### Cervical retraction

The retraction movement slightly increased the foramen area from C2/3 to C6/7 by 4.86 ± 1.26%, 3.88 ± 1.17%, 4.65 ± 2.35%, 2.81 ± 3.82%, and 2.20 ± 2.34%, respectively, as compared to the neutral position before treatment (all *p* > 0.001). After the treatment, the retraction movement increased the foramen area from C2/3 to C6/7 by 13.51 ± 3.85%, 9.16 ± 3.12%, 10.12 ± 3.74%, 6.63 ± 5.48%, and 7.99 ± 2.11%, respectively, as compared to the neutral position (*p* = 0.006 ~ 0.014). For the comparison before and after therapeutic exercise sessions, the area of the intervertebral foramen in the cervical retraction movement significantly increased after treatment in all segments (*p* = 0.002 ~ 0.036) with the exception of C5/6 (*p* = 0.071).

### Cervical lateral flexion toward the affected side

The active cervical lateral flexion toward the affected side decreased the area of the intervertebral foramen from C2/3 to C6/7 by 14.70 ± 3.12%, 12.22 ± 3.08%, 9.66 ± 2.85%, 10.24 ± 3.04%, and 6.55 ± 1.65%, respectively, as compared to the neutral position before treatment (*p* = 0.001 ~ 0.041) (Fig. 3c).

After the treatment, active cervical lateral flexion toward the affected side decreased the area of the intervertebral foramen from C2/3 to C6/7 by 21.25 ± 4.75%, 18.72 ± 3.88%, 15.16 ± 3.45%, 15.05 ± 3.94%, and 12.07 ± 2.34%, respectively, as compared to the neutral position (all *p* < 0.001). For the comparison before and after the therapeutic exercise sessions, the area of intervertebral foramen in terms of cervical lateral flexion toward the affected side significantly decreased to a greater extent after treatment (*p* = 0.005 ~ 0.026).

### Cervical lateral flexion away from the affected side

The lateral flexion away from the affected side increased the foramen area from C2/3 to C6/7 by 8.34 ± 2.88%, 7.91 ± 2.65%, 8.29 ± 3.12%, 5.77 ± 2.16%, and 6.49 ± 3.42%, respectively, before treatment (*p* = 0.003 ~ 0.026 for C2/3 and C4/5; *p* > 0.05 for C5/6 and C6/7). After the treatment, lateral flexion away from the affected side increased the foramen area from C2/3 to C6/7 by 15.07 ± 3.97%, 14.69 ± 4.03%, 14.02 ± 4.34%, 10.25 ± 3.71%, and 10.19 ± 5.23%, respectively, as compared to the neutral position (*p* = 0.004 ~ 0.014). The area of the intervertebral foramen during cervical lateral flexion away from the affected side significantly increased after treatment in all segments (*p* = 0.007 ~ 0.046) with the exception of C6/7 (*p* = 0.108).

The mean difference and 95% confidence intervals of the intervertebral foramen area before and after spinal therapeutic exercise movements, including cervical flexion-extension, protrusion-retraction, and lateral flexion toward or away from the affected side were summarized in Table 2.

**Table 2:** The mean difference and 95% confidence intervals of the intervertebral foramen area before and after spinal therapeutic exercise movements, including cervical flexion-extension, protrusion-retraction, and lateral flexion toward or away from the affected side. (* indicates with statistical difference)

**Level**	**Means Difference across Flexion**	**Lower limit of 95% CI**	**Upper limit of 95% CI**	**Means Difference across Extension**	**Lower limit of 95% CI**	**Upper limit of 95% CI**
C2/3	8.67%^*^	3.52%	13.81%	−9.18%^*^	−18.50%	0.14%
C3/4	6.60%^*^	−0.46%	13.66%	−0.69%	−10.30%	8.92%
C4/5	2.87%	−6.66%	12.39%	−5.12%^*^	−13.30%	3.05%
C5/6	2.91%	−6.94%	12.76%	−1.58%	−10.67%	7.51%
C6/7	5.02%^*^	0.20%	9.84%	−4.59%	−15.47%	6.29%
	**Means Difference across Protrusion**	**Lower limit of 95% CI**	**Upper limit of 95% CI**	**Means Difference across Retraction**	**Lower limit of 95% CI**	**Upper limit of 95% CI**
C2/3	−11.73%^*^	3.52%	13.81%	8.66%^*^	3.52%	13.81%
C3/4	−7.77%^*^	−16.60%	1.06%	5.28%^*^	1.07%	9.48%
C4/5	−9.13%^*^	−18.66%	0.40%	5.47%^*^	−0.50%	11.43%
C5/6	−10.39%	−19.26%	− 1.52%	3.82%^*^	−5.30%	12.93%
C6/7	−9.41%^*^	−15.78%	−3.04%	5.79%^*^	1.43%	10.15%
	**Means Difference across Lateral flexion toward**	**Lower limit of 95% CI**	**Upper limit of 95% CI**	**Means Difference across Lateral flexion away**	**Lower limit of 95% CI**	**Upper limit of 95% CI**
C2/3	−6.55%^*^	3.52%	13.81%	6.73%^*^	3.52%	13.81%
C3/4	− 6.50%^*^	−13.32%	0.32%	6.78%^*^	0.23%	13.33%
C4/5	−5.50%^*^	−11.68%	0.67%	5.73%^*^	−1.58%	13.05%
C5/6	−4.81%^*^	−11.65%	2.03%	4.48%^*^	−1.27%	10.23%
C6/7	−5.52%	−9.49%	−1.55%	3.71%^*^	−4.77%	12.18%

## Discussion

To provide additional insights into cervical biomechanics related to the patients with disc herniation, the dimensional changes in the intervertebral foramen of cervical spine during different active exercises were determined based on the validated radiographic protocol. This study is the first attempt to analyze the outcomes of active cervical therapeutic exercises on changes in the intervertebral foramen and after therapeutic exercise treatment sessions in patients with disc herniation. The areas of the intervertebral foramen generally increased in cervical flexion, retraction, and lateral flexion away from the affected side movements. In contrast, the areas of the intervertebral foramen generally decreased in cervical extension, protrusion, and lateral flexion toward the affected side movements. The 8-week therapeutic exercises led to the significant changes in the intervertebral foramen areas and improvement of patient’s status presence.

Considering the reliability tests in the present study, the ICCs and SEMs on identifying the vertebral landmarks in sequential videofluoroscopic images were in accordance with the cervical spinal researches [26, 29]. The error of locating vertebral landmarks was also comparable to the findings of Lee et al. [30]. The low radiation, reliable videofluoroscopic technique, and real-time visualization of vertebral segments is considered feasible in clinical and research applications. The minimal detectable change (MDC) for the measurement error in the present study ranged from 0.378 mm^2^ (0.828%) to 0.994 mm^2^ (2.174%) with an average of 0.797 mm^2^ (1.745%). This results indicated that the measurement for the active cervical therapeutic exercises on changes in the intervertebral foramen in patients with disc herniation with an error of approximately 2.2%. The study demonstrated that the meaningful statistical significance by the percentage (%) changes of the intervertebral foramen area before and after spinal therapeutic exercise movements exceeded the minimal detectable change (MDC) (Table 1).

The results of the present study showed that active cervical flexion increased the area of the patients’ intervertebral foramen from C2/3 to C6/7, and the extension exercise reduced the foramen area from C2/3 to C6/7. Our findings were similar to the reports of Yoo and colleagues [31]. They tested five fresh frozen adult human cadaver cervical spines (C2-T1) with combinations of flexion-extension and compared the results with the foraminal diameter at the neutral position. The neural foramen diameters increased significantly by 8 and 10% at 20 degrees and 30 degrees of flexion, respectively. Conversely, the neural foramen diameters underwent significant reductions of 10 and 13% at 20 degrees and 30 degrees of extension, respectively.

Although many spinal patients have demonstrated clinically meaningful improvements in pain relief and functional recovery following spinal exercises, the influences of dynamic positional changes in the head and neck on the dimensions of the neural foramen of the spine have not been well documented. Dynamic evaluations of changes in the intervertebral foramen during active therapeutic exercise are crucial for the understanding the cervical biomechanics and treating cervical disc herniation disorders. Cervical retraction exercise is a commonly prescribed therapeutic exercise for correction of forward head posture and restoration of extension of the cervical range to relieve disc pressure [19, 28, 32]. Lentell and colleagues described the cervical biomechanical relationship in healthy individuals by making a comparison in a neutral cervical spine position and a static retracted position using magnetic resonance imaging [33]. Their findings suggested that cervical retraction did not promote positional stenosis of the intervertebral foramen in a healthy neck. The major drawback of this previous study however was that the subjects were passively positioned in a supine position inside a magnet evaluation field, and this position did not reflect a real situation where the subjects actively performed the exercises or were physically examined in the upright position. Our subjects demonstrated similar findings when performing an active retraction exercise and increased the foramen area from C2/3 to C6/7 by 6.63–13.51%. This scenario may verify the efficacy of the retraction exercise for relieving the nerve compression within an intervertebral foramen [15, 16]. Although there is not much dynamic imaging evidence indicating this phenomenon, these previous findings and our results coincidentally suggest that therapeutic intervention methods may normalize the posture to physiological reference ranges and may thus be effective in the treatment of patients with neck or back pain.

In contrast, active cervical protrusion movement decreased the area of the intervertebral foramen in the disc-herniated patients under consideration in this study. Cervical protrusion movement resembles the forward head posture, the most common cervical postural fault in the sagittal plane that is found at different levels of severity in almost all populations. Some previous studies have demonstrated a correlation between forward head posture and neck pain [3, 34–36]. Smith and associates investigated the impact of cervical deformities, including forward head posture and upper thoracic kyphosis, on the anatomy of the cervical neural foramen using human cervical spine specimens [34]. They found that the area of the cervical neural foramens may decrease with increases in upper thoracic kyphosis, with greater reductions occurring in the lower cervical spine. In patients with such cervical deformities, cervical retraction exercises are commonly prescribed as the therapeutic strategy for the correction of forward head posture and restoration of the extension of the cervical range and length of the neck muscle. The exercise program is designed for patients to learn a new postural position, restore the original muscle length-tension relationships, restore normal joint mobility, and restore normal body balance [37, 38].

Generally, the present study provided dynamic evidence indicating changes to the intervertebral foramen during multi-directional exercises of the cervical spine. Based on the dynamic radiographic imaging analyses, the exercise outcomes leading to increases in the area of cervical neural foramen as high as 13% were obtained by having patients perform cervical flexion and retraction exercises. The findings of this study also indicate that awkward posture or improper movement patterns of the cervical spine may possibly reduce the area of the intervertebral foramen and consequently cause neck pain and nerve root entrapment. Although this study provides scientific evidence of changes in the dynamic intervertebral foramen during cervical movements, and the findings suggest clinical merits for encouraging or prohibiting some directions of neck movement as well, some limitations regarding the study design and the experimental processes still should be noted. The symptom duration of the disc-herniated patients was 3.4 months before intervention in the present study. The pain or numbness location were over neck, shoulder girdle, interscapular and forearm areas. Due to the medium- and long-term suffering of neck and arm pain, the patients did not vary the pain status and intensity much before the participation of this study. The mean visual analogue scale and neck disability index of these patients significantly decreased after the therapeutic exercise intervention. However, the study limitation needs to be concerned about spontaneous remission of symptoms from disc-herniation and therefore the present results need carefully interpreted. On the other hand, the use of videofluoroscopy to record and analyze the intervertebral foramen dimensions may have resulted in spatial information bias in 3D. Furthermore, the different cervical movement starting positions and variations in habitual control of cervical motion among each participant could possibly lead to variations in motion. A quantitative analysis of the intervertebral foramen may be employed to diagnose translation abnormalities such as hypomobility or hypermobility. Although the reliability and measurement errors are considered to be acceptable [39], the results of the present study must be interpreted with caution because of the limits related to enrolled subject numbers. Moremore, the present study focused on disc-herniated patients, future research should expand the subject groups across different spinal problems and ages and may reveal more complicated or even compensatory movements related to spinal impairments. Although the cervical therapeutic exercise was performed to the end range with less than 6/10 in visual analogue scale and without symptom peripheralization, the more extensive study to need to be conducted to understand if the changes in foramen areas relate to the pain intensity or to clinical improvements.

## Conclusions

The dimensional changes in the intervertebral foramen of the cervical spine during different active exercises were first described based on the validated radiographic protocol. The 8-week therapeutic exercises led to the significant changes in the intervertebral foramen areas and improvement of patient’s status presence.

## Data Availability

The research group is still working on conducting other analyses based on the current experimental data set of this study. However, parts of the datasets should be available from the corresponding author after approving by the Institutional Review Board on reasonable request.

## Abbreviations

ICC: Intraclass Correlation Coefficient
SEM: Standard Error of Measurement
MDC: Minimal Detectable Change
SPSS: Statistical Package for the Social Sciences

## References

[1] Ariëns GA, Bongers PM, Douwes M, Miedema MC, Hoogendoorn WE, van der Wal G, Bouter LM, van Mechelen W (2001). Are neck flexion, neck rotation, and sitting at work risk factors for neck pain? Results of a prospective cohort study. Occup Environ Med.

[2] Balogh I, Arvidsson I, Björk J, Hansson G, Ohlsson K, Skerfving S, Nordander C (2019). Work-related neck and upper limb disorders - quantitative exposure-response relationships adjusted for personal characteristics and psychosocial conditions. BMC Musculoskelet Disord.

[3] Nordander C, Hansson G, Ohlsson K, Arvidsson I, Balogh I, Strömberg U, Rittner R, Skerfving S: Exposure-response relationships for work-related neck and shoulder musculoskeletal disorders--Analyses of pooled uniform data sets. Appl Ergon 2016, 55:70–84. 10.1016/j.apergo.2016.01.010 26995038

[4] Jahre H, Grotle M, Smedbråten K, Dunn KM, Øiestad BE (2020). Risk factors for non-specific neck pain in young adults. A systematic review. BMC Musculoskelet Disord.

[5] Chan SC, Ferguson SJ, Gantenbein-Ritter B (2011). The effects of dynamic loading on the intervertebral disc. Eur Spine J.

[6] Komeili A, Rasoulian A, Moghaddam F, El-Rich M, Li LP (2021). The importance of intervertebral disc material model on the prediction of mechanical function of the cervical spine. BMC Musculoskelet Disord.

[7] Izzo R, Popolizio T, D'Aprile P, Muto M (2015). Spinal pain. Eur J Radiol.

[8] Li Y, Fredrickson V, Resnick DK (2015). How should we grade lumbar disc herniation and nerve root compression? A systematic review. Clin Orthop Relat Res.

[9] Griegel-Morris P, Larson K, Mueller-Klaus K, Oatis CA (1992). Incidence of common postural abnormalities in the cervical, shoulder, and thoracic regions and their association with pain in two age groups of healthy subjects. Phys Ther.

[10] Takahashi N, Yabuki S, Aoki Y, Kikuchi S (2003). Pathomechanisms of nerve root injury caused by disc herniation: an experimental study of mechanical compression and chemical irritation. Spine..

[11] Balkovec C, McGill S (2012). Extent of nucleus pulposus migration in the annulus of porcine intervertebral discs exposed to cyclic flexion only versus cyclic flexion and extension. Clin Biomech.

[12] Callaghan JP, McGill SM (2001). Intervertebral disc herniation: studies on a porcine model exposed to highly repetitive flexion/extension motion with compressive force. Clin Biomech.

[13] Pugely AJ, Ries Z, Gnanapragasam G, Gao Y, Nash R, Mendoza-Lattes SA (2017). Curve characteristics and Foraminal dimensions in patients with adult scoliosis and radiculopathy. Clin Spine Surg.

[14] Loch-Wilkinson TJ, Izatt MT, Labrom RD, Askin GN, Pearcy MJ, Adam CJ (2016). Morphometric analysis of the thoracic intervertebral foramen osseous anatomy in adolescent idiopathic scoliosis using low-dose computed tomography. Spine Deform.

[15] Lee JH, Choi KH, Kang S, Kim DH, Kim DH, Kim BR, Kim W, Kim JH, Do KH, Do JG (2019). Nonsurgical treatments for patients with radicular pain from lumbosacral disc herniation. Spine J.

[16] Żurawski A, Kiebzak W, Zmyślna A, Pogożelska J, Kotela I, Kowalski TJ, Śliwiński Z, Śliwiński G (2019). Efficacy of the use of the McKenzie and Vojta methods to treat discopathy-associated syndromes in the pediatric population. Int J Occup Med Environ Health.

[17] Wetzel FT, Donelson R (2003). The role of repeated end-range/pain response assessment in the management of symptomatic lumbar discs. Spine J.

[18] Long A, Donelson R, Fung T (2004). Does it matter which exercise? A randomized control trial of exercise for low back pain. Spine (Phila Pa 1976).

[19] Busanich BM, Verscheure SD (2006). Does McKenzie therapy improve outcomes for back pain?. J Athl Train.

[20] Chien JJ, Bajwa ZH (2008). What is mechanical back pain and how best to treat it?. Curr Pain Headache Rep.

[21] Fernández-de-Las-Peñas C (2009). Interaction between trigger points and joint Hypomobility: a clinical perspective. J Man Manip Ther.

[22] Edmond SL, Werneke MW, Young M, Grigsby D, McClenahan B, Harris G, McGill T (2020). Cognitive behavioural interventions, and function and pain outcomes among patients with chronic neck pain managed with the McKenzie approach. Musculoskeletal Care.

[23] Lam OT, Dumas JP, Simon CB, Tousignant-Laflamme Y (2018). McKenzie mechanical syndromes coincide with biopsychosocial influences, including central sensitization: a descriptive study of individuals with chronic neck pain. J Man Manip Ther.

[24] Berthelot JM, Delecrin J, Maugars Y, Passuti N (2007). Contribution of centralization phenomenon to the diagnosis, prognosis, and treatment of diskogenic low back pain. Joint Bone Spine.

[25] Fujiwara A, An HS, Lim TH, Haughton VM (2001). Morphologic changes in the lumbar intervertebral foramen due to flexion-extension, lateral bending, and axial rotation: an in vitro anatomic and biomechanical study. Spine (Phila Pa 1976).

[26] Kitagawa T, Fujiwara A, Kobayashi N, Saiki K, Tamai K, Saotome K (2004). Morphologic changes in the cervical neural foramen due to flexion and extension: in vivo imaging study. Spine (Phila Pa 1976).

[27] Koo TK, Li MY (2016). A guideline of selecting and reporting Intraclass correlation coefficients for reliability research. J Chiropr Med.

[28] Jette AM, Tao W, Norweg A, Haley S (2007). Interpreting rehabilitation outcome measurements. J Rehabil Med.

[29] Wang X, Lindstroem R, Carstens NPB, Graven-Nielsen T (2017). Cervical spine reposition errors after cervical flexion and extension. BMC Musculoskelet Disord.

[30] Lee SW, Wong KWN, Chan MK, Yeung HM, Chiu JLF, Leong JCY (2002). Development and validation of a new technique for assessing lumbar spine motion. Spine.

[31] Yoo JU, Zou D, Edwards WT, Bayley J, Yuan HA (1992). Effect of cervical spine motion on the neuroforaminal dimensions of human cervical spine. Spine (Phila Pa 1976).

[32] May S, Gardiner E, Young S, Klaber-Moffett J (2008). Predictor variables for a positive Long-term functional outcome in patients with acute and chronic neck and Back pain treated with a McKenzie approach: a secondary analysis. J Man Manip Ther..

[33] Lentell G, Kruse M, Chock B, Wilson K, Iwamoto M, Martin R (2002). Dimensions of the cervical neural foramina in resting and retracted positions using magnetic resonance imaging. J Orthop Sports Phys Ther.

[34] Smith ZA, Khayatzadeh S, Bakhsheshian J, Harvey M, Havey RM, Voronov LI, Muriuki MG, Patwardhan AG (2016). Dimensions of the cervical neural foramen in conditions of spinal deformity: an ex vivo biomechanical investigation using specimen-specific CT imaging. Eur Spine J.

[35] Mahmoud NF, Hassan KA, Abdelmajeed SF, Moustafa IM, Silva AG (2019). The relationship between forward head posture and neck pain: a systematic review and Meta-analysis. Curr Rev Musculoskelet Med.

[36] Kim EK, Kim JS (2016). Correlation between rounded shoulder posture, neck disability indices, and degree of forward head posture. J Phys Ther Sci.

[37] Bronfort G, Haas M, Evans RL, Bouter LM (2004). Efficacy of spinal manipulation and mobilization for low back pain and neck pain: a systematic review and best evidence synthesis. Spine J.

[38] Young IA, Michener LA, Cleland JA, Aguilera AJ, Snyder AR (2009). Manual therapy, exercise, and traction for patients with cervical radiculopathy: a randomized clinical trial. Phys Ther.

[39] Wu SK, Jou JY, Lee HM, Chen HY, Su FC, Kuo LC (2015). The reproducibility comparison of two intervertebral translation measurements in cervical flexion-extension. Spine J.

